# Incidental functional paraganglioma of the celiac space discovered during breast cancer staging: a case report

**DOI:** 10.1093/jscr/rjaf991

**Published:** 2025-12-17

**Authors:** Sabrillah Echiguer, Chemsdine Echiguer, Zaynab Laoufi, Soumya El Graini, Yassine El Bouazizi, Zakaria El Mouatassim, Oumayma Lahnaoui, Youssef Omor, Rachida Latib, Mohammed Anass Majbar, Amine Souadka, Amine Benkabbou

**Affiliations:** Department of Surgical Oncology, National Institute of Oncology, Faculty of Medicine and Pharmacy, Mohammed V University in Rabat, Morocco; Department of Medical Biology, Faculty of Medicine and Pharmacy, Mohammed V University in Rabat, Morocco; Department of Cardiology, Faculty of Medicine and Pharmacy, Mohammed V University in Rabat, Morocco; Department of Radiology, National Institute of Oncology, Faculty of Medicine and Pharmacy, Mohammed V University in Rabat, Morocco; Department of Surgical Oncology, National Institute of Oncology, Faculty of Medicine and Pharmacy, Mohammed V University in Rabat, Morocco; Department of Surgical Oncology, National Institute of Oncology, Faculty of Medicine and Pharmacy, Mohammed V University in Rabat, Morocco; Department of Surgical Oncology, National Institute of Oncology, Faculty of Medicine and Pharmacy, Mohammed V University in Rabat, Morocco; Department of Radiology, National Institute of Oncology, Faculty of Medicine and Pharmacy, Mohammed V University in Rabat, Morocco; Department of Radiology, National Institute of Oncology, Faculty of Medicine and Pharmacy, Mohammed V University in Rabat, Morocco; Department of Surgical Oncology, National Institute of Oncology, Faculty of Medicine and Pharmacy, Mohammed V University in Rabat, Morocco; Department of Surgical Oncology, National Institute of Oncology, Faculty of Medicine and Pharmacy, Mohammed V University in Rabat, Morocco; Department of Surgical Oncology, National Institute of Oncology, Faculty of Medicine and Pharmacy, Mohammed V University in Rabat, Morocco

**Keywords:** functional paraganglioma, celiac space, breast cancer, incidentaloma, catecholamine crisis

## Abstract

Paragangliomas are rare neuroendocrine tumors arising from extra-adrenal chromaffin cells. Although often silent, functional paragangliomas may present with catecholamine hypersecretion and life-threatening complications. Their incidental discovery during cancer staging is exceptionally uncommon and poses unique therapeutic challenges. We report the case of an 82-year-old woman presenting with breast cancer, in whom staging investigations revealed a 6-cm functional paraganglioma of the celiac space. A multidisciplinary team prioritized paraganglioma resection before oncologic breast surgery. Preoperative alpha-adrenergic blockade was administered for 15 days. Surgery was complicated by catecholamine surges requiring intensive hemodynamic and critical care support. Postoperative recovery was uneventful. Histopathological analysis confirmed paraganglioma, and the patient was subsequently referred back for breast surgery. This case illustrates the rare incidental discovery of a hormonally active celiac paraganglioma during breast cancer staging. It highlights the need for multidisciplinary planning and emphasizes the role of perioperative critical care in optimizing outcomes of functional paragangliomas.

## Introduction

Incidental findings during cancer staging may reveal unexpected conditions that alter the initial therapeutic plan. In our case, routine breast cancer staging identified a functional paraganglioma in the celiac space.

Paragangliomas are rare neuroendocrine tumors arising from extra-adrenal chromaffin cells, belonging to the pheochromocytoma–paraganglioma (PPGL) spectrum [1]. Although often silent [2], hormonally active forms can cause life-threatening complications [3], making surgical resection the cornerstone of management [4].

To our knowledge, no functional paraganglioma associated with breast cancer has been previously reported in the literature. This case illustrates the diagnostic and therapeutic challenges of this unusual association and highlights the importance of multidisciplinary coordination.

## Case report

We report the case of an 82-year-old woman with a 10-year history of arterial hypertension on medical treatment, who presented to her gynecologist with a right breast mass. Clinical examination revealed a mass measuring approximately 2 × 3 cm at the junction of the upper quadrants of the right breast, without skin changes or palpable lymphadenopathy. The contralateral breast and general examination were normal.

Mammography and breast ultrasound confirmed a suspicious right breast lesion measuring 20 × 31 mm, categorized as BI-RADS breast imaging reporting and data system (BI-RADS) 5, with a 10 × 5 mm right axillary lymph node. Core needle biopsy revealed an invasive carcinoma of no special type, with estrogen receptor positivity in 90% of tumor cells, progesterone receptor positivity in 5%, and human epidermal growth factor receptor 2 (HER2) negativity assessed by immunohistochemistry (IHC), with a score of 0.

**Figure 1:**
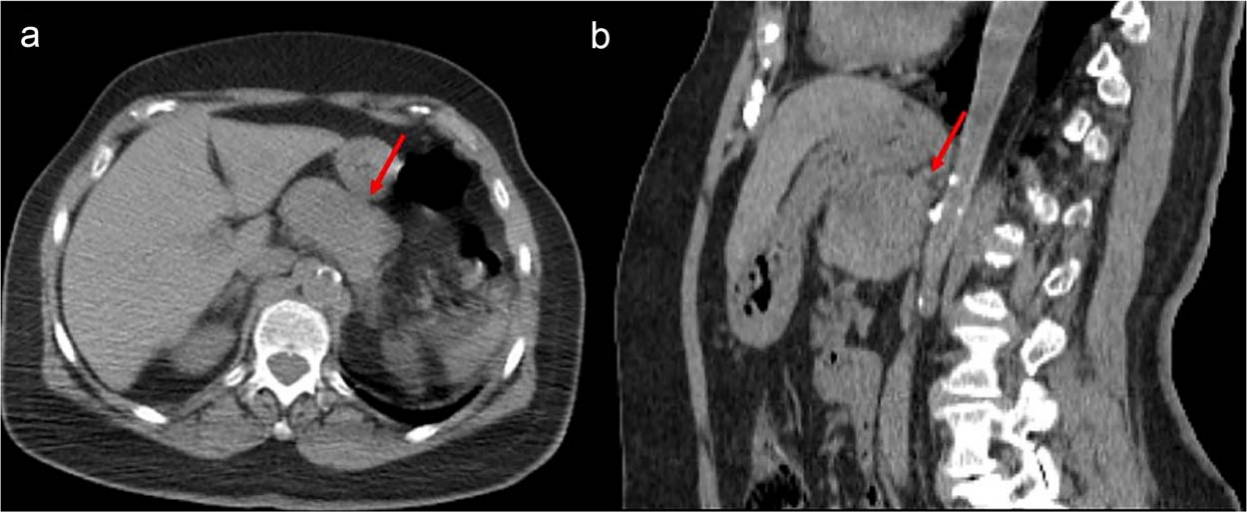
Unenhanced CT scan, axial (a), and sagittal reconstruction (b), revealing a well-defined oval mass with regular contours and soft-tissue density, closely contacting the posterior wall of the stomach and appearing to occupy the omental bursa. The lesion does not invade adjacent structures.

**Figure 2:**
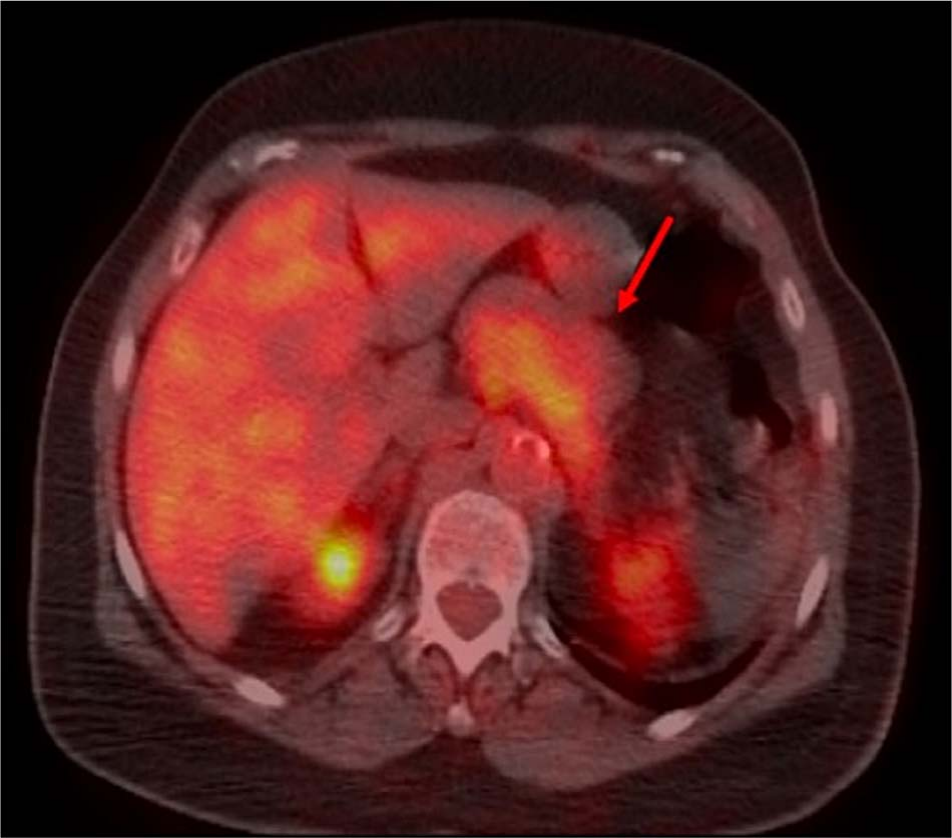
FDG PET scan demonstrating hypermetabolism of the mass.

**Figure 3:**
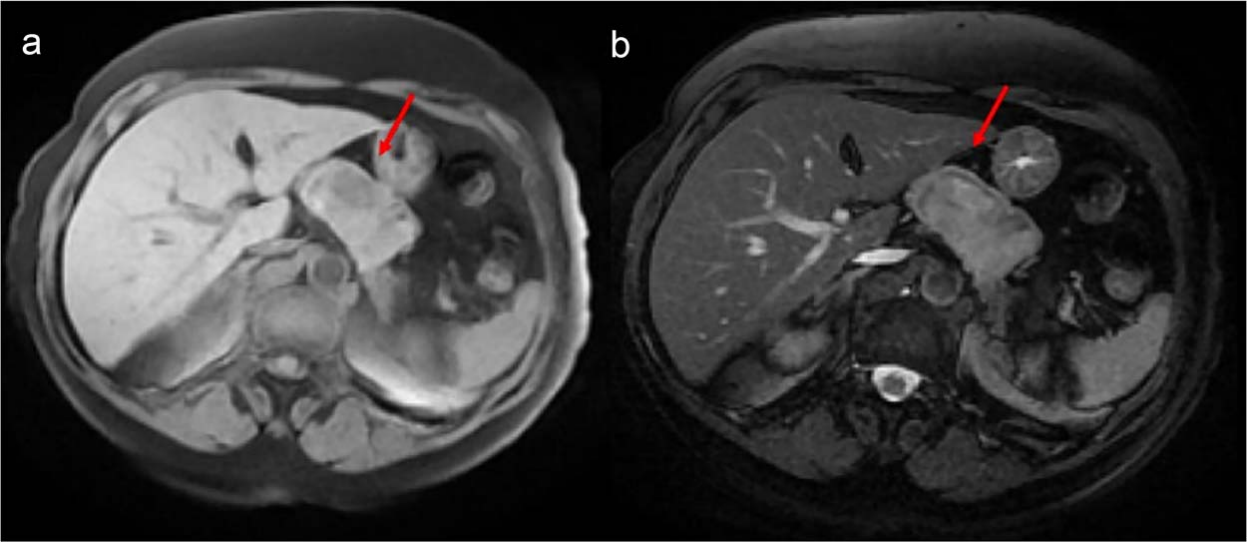
Abdominal MRI showing a heterogeneous intermediate T1 signal mass (a) with slight hyperintensity on T2-weighted images (b), without fatty components, closely contacting the stomach but distant from the liver.

A thoraco-abdominopelvic computed tomography (CT) scan performed for staging revealed a well-defined, homogeneous soft-tissue mass anterior to the celiac trunk, superior to the pancreas, and medial to the stomach (Fig. 1). A positron emission tomography (PET) scan confirmed the previously known hypermetabolic retroareolar lesion of the right breast and revealed a large hypermetabolic mass in the celiac–mesenteric region, measuring 63 × 35 × 50 mm (Fig. 2). An abdominal MRI characterized the mass as a well-defined 6-cm lesion with mixed solid and cystic signal intensity, closely related to the celiac trunk, pancreas, stomach, and left adrenal gland (Fig. 3). Biochemical testing showed elevated urinary normetanephrine and metanephrine levels, consistent with catecholamine hypersecretion.

**Figure 4:**
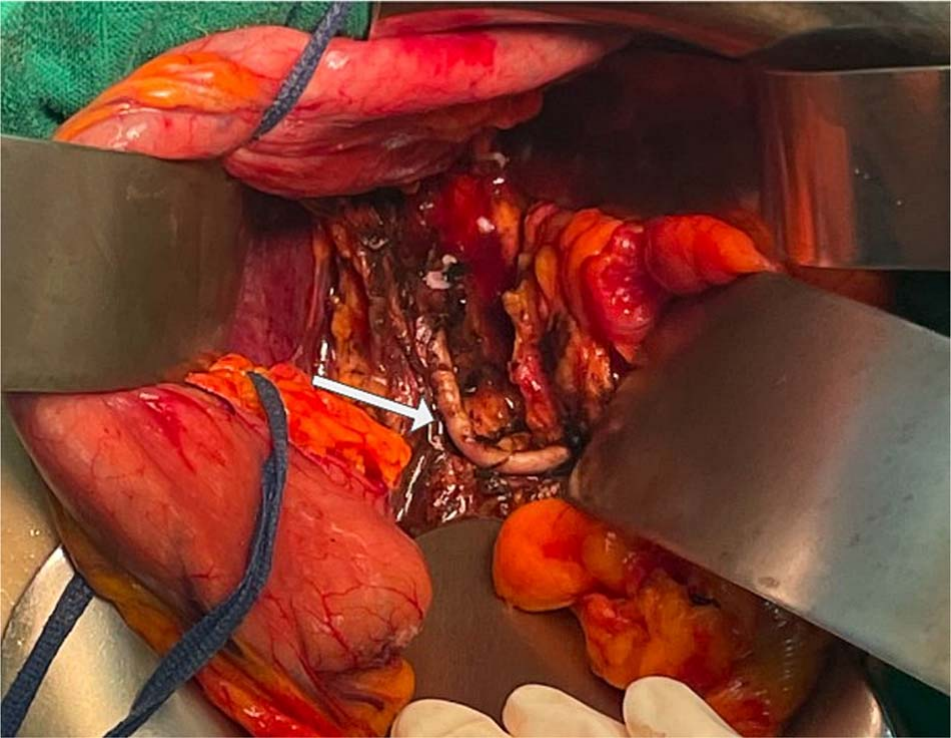
Intraoperative view after complete resection of the mass, showing displacement of the splenic artery (arrow).

**Figure 5:**
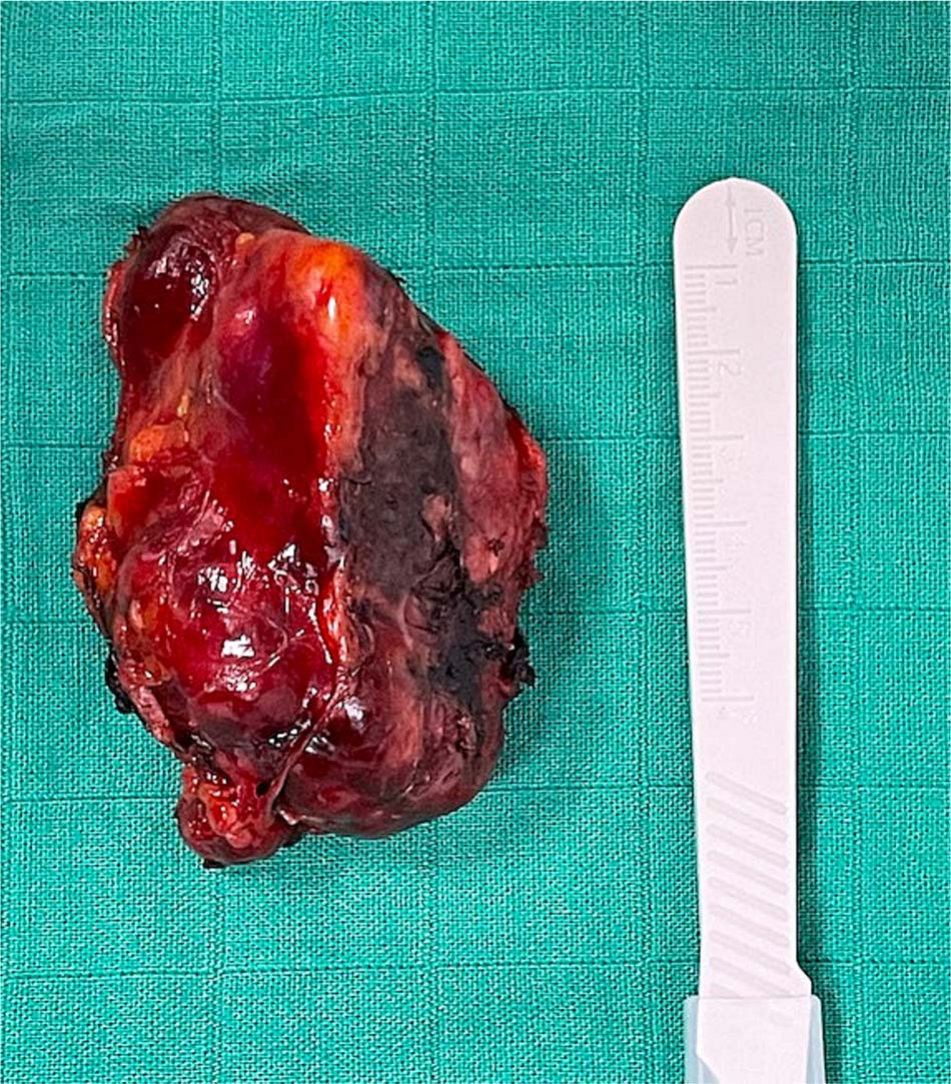
Surgical specimen.

The patient was referred to our center, where a multidisciplinary team meeting recommended paraganglioma resection before breast surgery. Preoperative preparation consisted of 15 days of alpha-adrenergic blockade. Given the localization, size, and anatomical relationships of the mass, we opted for open surgery via laparotomy. Intraoperative exploration revealed a 6-cm mass located anterior to the celiac trunk, adherent to the superior surface of the pancreas and displacing the splenic artery. Mobilization of the mass triggered catecholamine surges causing episodes of hypertension and tachycardia, which were managed by the anesthesia team. Dissection of the mass from the splenic artery and the superior surface of the pancreas was technically demanding (Fig. 4). After complete removal of the mass (Fig. 5), the patient developed hypotension requiring norepinephrine support. She was transferred to the intensive care unit postoperatively, successfully weaned from norepinephrine, and extubated the same day.

On postoperative day 1, the patient was transferred back to the surgical ward. Her recovery was uneventful, and she was discharged on postoperative day 5. Histopathological examination confirmed the diagnosis of paraganglioma. The patient was referred to her gynecologist for breast surgery. The multidisciplinary team will decide, after breast surgery, on the modalities of surveillance for both the breast cancer and the paraganglioma.

## Discussion

Paragangliomas are rare tumors accounting for 15%–20% of all PPGLs, with an incidence of 0.5 cases per million annually [5]. Most paragangliomas are diagnosed between the third and fifth decades of life [6]; however, they can occur at any age, as illustrated by our patient, who was diagnosed at 80 years old. According to the WHO classification [7], pheochromocytomas originate in the adrenal medulla while paragangliomas are extra-adrenal and can be located in the abdomen, thorax, pelvis, and neck. Head and neck paragangliomas generally do not produce catecholamines because they arise from parasympathetic ganglia [4], whereas most tumors derived from sympathetic ganglia are found in the abdomen and secrete excess catecholamines [3], as was the case in our patient.

The traditional “rule of 10%” for PPGLs, which suggested that 10% of cases were associated with a positive family history, 10% were malignant, 10% were bilateral, and 10% had an extra-adrenal origin, has been challenged by recent advances in diagnostic techniques and the identification of associated germline mutations [4]. In this context, genetic testing is recommended for all patients with PPGLs, not only for familial screening, but also for both diagnosis and prognostication [1, 8]. Catecholamine hypersecretion is the most severe and life-threatening feature, typically presenting with headache, palpitations, sweating, and episodic hypertension in about one third of patients, while others show atypical symptoms that may delay diagnosis. In rare cases, excessive catecholamine release can cause hypertensive crises, potentially leading to heart attack, stroke, heart failure, or even cardiovascular collapse [3]. Biochemical testing remains the cornerstone of PPGL diagnosis, aiming to confirm catecholamine hypersecretion. Current guidelines recommend assessing plasma free or urinary fractionated metanephrines [1].

Surgical removal is the standard treatment for PPGL [4]. Open surgery is recommended for paragangliomas, but laparoscopic resection can be performed for small, noninvasive tumors located in surgically favorable locations [1]. Intraoperative manipulation can induce a massive release of catecholamines leading to hypertensive crisis, arrhythmias, ischemia, pulmonary edema, or stroke [9]. Preoperative alpha-adrenergic blockade for 7 to 14 days is recommended to stabilize hemodynamics and prevent hypotension after tumor removal [1]. In our patient, although adequate alpha-adrenergic blockade was administered, she developed intraoperative hemodynamic instability requiring norepinephrine after resection. Follow-up protocols are not standardized and should be individualized based on symptoms, metanephrine levels, and imaging findings [10].

In conclusion, the incidental discovery of a hormonally active paraganglioma during breast cancer staging posed a unique therapeutic dilemma. By prioritizing paraganglioma resection before breast surgery, the multidisciplinary team successfully managed a rare and high-risk clinical scenario, offering a practical approach to similar future cases. In this context, it is worth emphasizing that paragangliomas, although rare and often silent, may have life-threatening consequences and should be considered in atypical clinical presentations.
